# Association between corrected serum calcium levels after dialysis and post-dialysis fatigue risk: a hospital-based case–control study

**DOI:** 10.1186/s40001-023-01131-5

**Published:** 2023-05-15

**Authors:** Ping Xiao, Zhi-Hong Wang, Yan Lu, Shuang Zhang, Yu-Xin Jin, Xin Liu, Zhen-Li Jiang, Shu-Xin Liu

**Affiliations:** 1 Department of Nephrology, Dalian Municipal Central Hospital, No.826, Xinan Road, Dalian, 116033 Liaoning People’s Republic of China; 2 Key Laboratory of Intelligent Blood Purification, Dalian City. Dalian Municipal Central Hospital, Dalian, China; 3 Department of Nephrology, Dalian Municipal Central Hospital affiliated with Dalian University of Technology, Dalian, China

**Keywords:** Maintenance hemodialysis, Post-dialysis fatigue, Serum calcium

## Abstract

**Objective:**

Post-dialysis fatigue (PDF) is an important problem in patients undergoing maintenance hemodialysis (MHD); however, evidence of the association between serum calcium after dialysis and the risk of PDF is limited and controversial. We explored this association among patients receiving MHD.

**Methods:**

We carried out a case–control study of patients in the dialysis unit of Dalian Municipal Central Hospital between December 2019 and January 2020, including 340 patients with PDF and 270 patients without PDF. PDF was assessed by a \validated self-administered questionnaire. Clinical variables were tested for multicollinearity using variance inflation factor analysis. Corrected serum calcium levels were categorized into three groups, with the lowest tertile used as the reference category. The odds ratios (ORs) and corresponding 95% confidence intervals (CIs) for PDF risk were estimated using an unconditional logistic regression model.

**Result:**

After adjusting for potential confounders, corrected serum calcium levels showed a significant positive association with the risk of PDF (OR_T3vs.T1_ = 1.61, 95% CI = 1.01–2.58). Notably, after stratification by age, corrected serum calcium was also positively associated with the risk of PDF in patients aged ≥ 65 years (OR_T3vs.T1_  = 4.25, 95% CI 1.66–11.46). Furthermore, a significant linear trend and interaction were also observed (*P* < 0.05).

**Discussion:**

Higher corrected serum calcium levels after dialysis might increase the risk of PDF among MHD patients. However, further studies are warranted to confirm these findings.

## Introduction

Post-dialysis fatigue (PDF) is a common and distressing symptom, described as feeling tired and needing to rest or sleep after a dialysis session [1]. Piper et al. defined fatigue as "a subjective feeling of tiredness that is influenced by circadian rhythms and can vary in unpleasantness, duration, and intensity" [2]. The prevalence of PDF is relatively high, and affects 60–97% of patients undergoing long-term renal replacement treatment [3]. Several studies have indicated that fatigue severely impairs physical and social functioning and may also be associated with lower quality of life and premature death in patients receiving maintenance hemodialysis (MHD) [4–6]. Furthermore, untreated fatigue in MHD patients might cause weakness, reduced physical and psychological energy, social isolation, and depression [7], and is thus an important factor affecting the prognosis of patients receiving MHD [8, 9]. However, although PDF is likely to be associated with both genetic and environmental factors, the underlying mechanisms are not fully understood. Several studies have suggested that PDF may be associated with socio-demographic factors (e.g., age, sex, race, employments status) and clinical variables (e.g., anemia, malnutrition, sleep disorders, secondary hyperparathyroidism), as well as psychological (e.g., anxiety, stress, depression) and dialysis-related factors [10–14]. It is therefore crucial to clarify the influence of clinical variables on PDF among these MHD patients.

Disorders of serum calcium metabolism are highly prevalent among patients with end-stage renal disease (ESRD) and are associated with poor clinical outcomes [15, 16]. However, studies on the association between serum calcium and PDF among MHD patients have been scarce and have often presented conflicting results [17–19]. For example, Bossola et al. found no association between serum calcium and PDF risk in ESRD patients receiving chronic hemodialysis (HD) [17], while Zuo et al. indicated that serum calcium was negatively associated with fatigue in patients on HD [18], and Karakan et al. found a positive association between serum calcium and overall fatigue score in patients undergoing chronic HD [19].

In light of the inadequate evidence regarding the relationship between serum calcium and the risk of PDF among MHD patients in China, we conducted a large hospital-based case–control study with confounding factor adjustment to identify the relationship between corrected serum calcium concentrations after dialysis and the risk of PDF among Chinese MHD patients. These findings could help to predict patients at risk of PDF and thus allow their timely treatment.

## Materials and methods

### Design and population

We performed a hospital-based case–control study of patients receiving MHD in the HD unit of Dalian Municipal Central Hospital from December 2019 to January 2020. The inclusion criteria were patients aged ≥ 18 years who had received of HD for ≥ 3 months, and who voluntarily agreed to participate in the study. Patients aged ≥ 80 years or with acute kidney injury or active malignancy were excluded. All eligible patients underwent a total of 12 h of double reverse osmosis water and standard bicarbonate dialysis three times a week. PDF was assessed according to the recommendations reported by Sklar et al. [10, 11]. Each patient was interviewed though written questionnaire during one regularly scheduled treatment and patients were considered to be suffering from PDF if they spontaneously replied positively to the open-ended question: "Do you feel fatigued after dialysis?". Conversely, control patients were patients from the same unit who did not report any feeling of PDF during the study period. All study protocols conformed to the principles of the Declaration of Helsinki and were approved by the institutional medical ethics committee of Dalian Municipal Central Hospital, and all study patients provided written informed consent.

### Data collection

Demographic variables including age, sex, dialysis duration, body mass index (BMI), comorbidities including diabetes mellitus, hypertension, residual renal function, daily exercise time and clinical variables including causes of ESRD were gathered by the self-administered questionnaire and from electronic record forms in the hospital information system of Dalian Municipal Central Hospital. The causes of ESRD were divided into five categories: diabetic nephropathy, glomerulonephritis, hypertensive benign renal arterioles, polycystic kidney disease, and other diseases. Dialysis duration was defined as the time between the first day of HD treatment and the first day of entry into the study. The definition of diabetes mellitus was a past medical history of diabetes or the use of glucose-lowering medication, while that of hypertension was having a medical record of hypertension or using anti-hypertensive drugs.

Blood specimens were taken from all included patients using standardized techniques before their mid-week MHD session in December 2019, and stored at – 80 ℃ for assay. A range of laboratory variables including serum concentrations of hemoglobin, albumin, urea nitrogen, phosphorus, calcium, C-reactive protein (CRP), pre-dialysis and post-dialysis sodium levels, magnesium, intact Parathyroid Hormone, ultrafiltration rate and single-pool Kt/V_urea_ (spKt/V) were measured using standard procedures. Corrected serum calcium was calculated as: calcium (mmol/L) + 0.024 (40 − albumin [g/L]). spKt/V [(clearance of urea × dialysis time)/patient’s total body water] was used to estimate dialysis dose. All data were obtained using standard laboratory procedures and an automatic analyzer.

### Statistical analysis

PDF was treated as a dichotomous dependent variable in the analysis. Corrected serum calcium acted as the main independent variable and was calculated as categorical variables based on tertiles of distribution in the control group, with the lowest tertile serving as the reference group. The normality of the data was evaluated using the Shapiro–Wilk test. Continuous variables were expressed as mean ± standard deviation (SD) or median (quartile 1-quartile 3), and intergroup comparisons were analyzed using one-way ANOVA tests for normally distributed data or the Mann–Whitney U test for non-normally distributed data. Categorical variables were expressed as numbers and percentages, and differences between two groups were examined using χ^2^ tests. Based on previous studies [17–19]and clinical experience, we applied three logistic regression models to calculate the odds ratios (ORs) and 95% confidence intervals (CIs). In model 1, we calculated crude ORs and 95% CIs without adjusting any covariates, model 2 was adjusted for sex, age (continuous, years), and dialysis duration (continuous, months), and model 3 was additionally adjusted for BMI (continuous, kg/m^2^), daily exercise time (no/less than 1 h/1–2 h/ greater than 2 h), residual renal function (yes or no), hemoglobin (continuous, g/L), albumin (continuous, g/L), urea nitrogen (continuous, mmol/L), phosphorus (continuous, mmol/l), CRP (continuous, mg/L), spKt/V, hypertension (yes/no), diabetes (yes/no). Subgroup analyses were used to assess the potential effect modification by age (< 65 years, ≥ 65 years). Linear trend tests were performed by assigning the median corrected serum calcium level and treating it as a continuous variable in the logistic regression models. Multicollinearity among clinical variables was examined using variance inflation factors. Potential interaction of corrected serum calcium with the stratifying variable was assessed by adding cross-product terms to the multivariable logistic regression models. All analyses were carried out using SAS^®^ version 9.4 (SAS Institute Inc., Cary, NC, USA). Statistical significance was set at *P* < 0.05 and based on two-sided tests.

## Results

Eight of 348 potential cases and eight of 278 potential controls had missing information or unreasonable values for some variables and were therefore excluded. A total of 340 cases and 270 controls thus replied to the open-ended question and were included the study. The participation rates were 98% among cases and 97% among controls. The distribution of general characteristics among the cases and controls is presented in Table 1. The main analysis included 340 cases with PDF and 270 controls. The median age and mean corrected serum calcium were significantly higher in the cases compared with the controls. Conversely, patients with PDF had significantly lower albumin concentrations and less exercise time than the controls. There were no significant differences in any other variables between the two groups.

**Table 1:** General characteristics of patients in a case–control study of post-dialysis fatigue

Characteristic	Study patients (*n* = 610)	Fatigue (*n* = 340)	Non-Fatigue (*n* = 270)	*P*
Age, years	58 (47–66)	59 (49–67)	56 (46–64)	0.02
Male, *n* (%)	375 (61.48)	203 (59.71)	172 (63.70)	0.31
Time on dialysis, months	51(24–99)	50.5(25.5–101)	51.5 (23–95)	0.37
BMI (kg/m^2^)	23.5 (21–26.1)	23.80 (21.05–26.20)	23.20 (20.9–25.7)	0.17
Primary disease, *n* (%)				0.07
Diabetic nephropathy	202 (33.11)	116 (34.12)	86 (31.85)	
Glomerulonephritis	239 (39.18)	127 (37.75)	112 (41.48)	
Hypertensive nephropathy	99 (16.23)	65 (19.12)	34 (12.59)	
Polycystic kidney	33 (5.41)	13 (3.82)	20 (7.41)	
Others	37 (6.07)	19 (5.59)	18 (6.67)	
Residual renal function [yes, *n* (%)]	227 (37.21)	118 (34.71)	109 (40.37)	0.15
Hypertension [yes, *n* (%)]	453 (75.75)	250 (75.08)	203 (76.60)	0.66
Diabetes [yes, *n* (%)]	223 (37.29)	124 (37.24)	99 (37.36)	0.98
Daily exercise time *n* (%)				< 0.01
No	242 (39.67)	156 (45.88)	86 (31.85)	< 0.01
Less than 1 h	181 (29.67)	108 (31.76)	73 (27.04)	0.20
1–2 h	123 (20.16)	50 (14.71)	73 (27.04)	< 0.01
Greater than 2 h	64 (10.49)	26 (7.65)	38 (14.07)	0.01
UFR, ml/kg∙h				0.74
< 12.5	567 (92.95)	315 (92.65)	252 (93.33)	
≥ 12.5	43 (7.05)	25 (7.35)	18 (6.67)	
Hemoglobin, g/L	113 (102–122)	112.5 (100.5–122)	113 (104–121)	0.46
Albumin, g/L	41.9 (39.5–43.8)	41.5 (39.30–43.55)	42.2 (40–44)	0.01
Urea nitrogen, mmol/L	26.25 (22.65–30.03)	26.20 (22.75–30.11)	26.30 (22.40–30.01)	0.68
C-reactive protein, mg/L	3.14(3.14–7.71)	3.36 (3.14–7.74)	3.14 (3.14–7.62)	0.49
Phosphorus, mmol/L	1.99 (1.66–2.40)	1.99 (1.66–2.41)	1.99 (1.64–2.35)	0.68
Calcium, mmol/L	2.42 ± 0.12	2.43 ± 0.12	2.40 ± 0.13	< 0.01
Single-pool Kt/V_urea_	1.31 (1.17–1.47)	1.31 (1.17–1.47)	1.31 (1.18–1.47)	0.74
Pre-dialysis sodium, mmol/L	137.43 ± 3.41	137.34 ± 3.47	137.55 ± 3.35	0.44
Post-dialysis sodium, mmol/L	136.01 ± 2.29	136.07 ± 2.30	135.93 ± 2.28	0.46
Magnesium, mmol/L	1.09 (1.02–1.19)	1.10 (1.02–1.19)	1.09 (1.01–1.18)	0.57
iPTH, pg/mL	497.30 (278.10–803.90)	479.15 (274.45–804.75)	506.65 (280.80–797)	0.54

BMI, body mass index; UFR, ultrafiltration rate; iPTH, intact Parathyroid Hormone

Data are presented as means and standard deviations or medians [quartile 1, quartile 3] for continuous variables; and as frequencies and percentages for categorical variables

*P* values were determined with one-way ANOVA tests or Mann–Whitney *U* tests for continuous variables and chi-square test for categorical variables

All statistical tests are two sided

The estimated ORs and 95% CIs for the crude and multivariable logistic regression models of the association between tertiles of corrected serum calcium and PDF are shown in Table 2. Overall, compared with the lowest tertile of corrected serum calcium, a higher corrected serum calcium level was associated with an increased risk of PDF in the fully adjusted model (OR = 1.61, 95% CI = 1.01–2.58). However, no significant linerar trend was observed. In addition, the finding that the variance inflation values were < 5 in the linear regression model is considered to indicate the absence of multicollinearity among the variables (data not shown).

**Table 2:** Odds ratio and 95% confidence interval for post-dialysis fatigue by the corrected serum calcium levels

Variables	Fatigue (*N* = 340)	Non-fatigue (*N* = 270)	Model1^a^ OR (95% CI)	Model2^b^ OR (95% CI)	Model3^c^ OR (95% CI)
Total calcium, mmol/l					
T_1_ (< 2.33)	76	89	1.00 (ref)	1.00 (ref)	1.00 (ref)
T_2_ (2.33–2.46)	134	90	1.74 (1.16–2.62)	1.66 (1.10–2.52)	1.55 (1.00–2.42)
T_3_ (> 2.46)	130	91	1.67 (1.12–2.52)	1.70 (1.12–2.59)	1.61 (1.01–2.58)
*P* trend			0.021	0.019	0.061

CI, confidence interval; OR, odds ratio; T, tertiles; ref, reference

^a^Model 1: Crude model

^b^Model 2: Adjusted for age, gender and dialysis age

^c^Model 3: Adjusted for age, gender, dialysis age, body mass index, residual kidney function, hemoglobin, albumin, urea nitrogen, single-pool Kt/V_urea_, C-reactive protein, phosphorus, hypertension, diabetes and daily exercise time

The results of subgroup analyses stratified by age are shown in Table 3. When stratified by age, patients aged ≥ 65 years in the highest tertile of corrected serum calcium had a higher risk of PDF (OR = 4.25, 95% CI = 1.66–11.46). Furthermore, a linear trend (*P* trend < 0.05) and a significant interaction (*P*_interaction_ = 0.047) were also evident.

**Table 3:** Odds ratio (ORs) and 95% CIs for post-dialysis fatigue by the corrected serum calcium levels stratified by age

Total calcium, mmol/l	Fatigue (*N* = 340)	Non-fatigue (*N* = 270)	Model1^a^ OR (95% CI)	Model2^b^ OR (95% CI)	Model3^c^ OR (95% CI)	*P*_interaction_
< 65						
T_1_ (< 2.35)	61	67	1.00 (ref)	1.00 (ref)	1.00 (ref)	
T_2_ (2.35–2.47)	81	67	1.33 (0.83–2.14)	1.31 (0.81–2.11)	1.16 (0.69–1.95)	
T_3_ (> 2.47)	88	69	1.40 (0.88–2.24)	1.36(0.85-2.19)	1.09 (0.63–1.89)	
*P* trend			0.164	0.209	0.776	0.047
≥ 65						
T_1_ (< 2.32)	20	22	1.00 (ref)	1.00 (ref)	1.00 (ref)	
T_2_ (2.32–2.39)	26	22	1.30 (0.57–3.00)	1.23 (0.53–2.87)	1.84 (0.70–5.03)	
T_3_ (> 2.39)	64	23	3.06 (1.42–6.69)	2.86 (1.32–6.32)	4.25 (1.66–11.46)	
*P* trend			0.002	0.005	0.003	

CI, confidence interval; OR, odds ratio; T, tertiles; ref, reference

^a^Model 1: Crude model

^b^Model 2: Adjusted for gender and dialysis age

^c^Model 3: Adjusted for gender, dialysis age, body mass index, residual kidney function, hemoglobin, albumin, urea nitrogen, single-pool Kt/V_urea_, C-reactive protein, phosphorus, hypertension, diabetes and daily exercise time

## Discussion

In this hospital-based case–control study, we observed a significant positive relationship between corrected serum calcium after dialysis and the risk of PDF. Subgroup analyses indicated that the level of corrected serum calcium was associated with an increased risk of PDF among older patients (aged ≥ 65 years).

Previous studies found inconsistent results regarding the association between serum calcium levels and the risk of PDF. A cross-sectional survey in China by Zuo et al. indicated that serum calcium was negatively associated with fatigue in patients undergoing HD [18]. This apparent discrepancy with the current results might be due to differences in the methods of fatigue assessment, sample sizes, incorporated calcium form, and the covariates assessed. The previous study included 511 patients receiving HD and only explored the correlation between calcium and fatigue, without adjusting for any covariates. In addition, fatigue symptoms were evaluated using the Revised Piper Fatigue Scale in their study. A prospective multicenter study in Italy conducted by Bossola et al. found no association between serum calcium and PDF risk in ESRD patients receiving chronic HD [17]. This inconsistency with our findings might be attributable to differences in geographical locations and/or ethnicity, and their small sample size (only 271 HD patients). Nevertheless, another cross-sectional study by Karakan et al. in Turkey found a positive association between serum calcium and overall fatigue scores in patients undergoing chronic HD [19], consistent with the current results.

Although the present study found an association between corrected serum calcium levels after dialysis and the risk of PDF, the underlying biological mechanism remains unclear. Disturbances of calcium and phosphorus metabolism are common complications in HD patients, which could in turn activate the oxidative stress response and present a micro-inflammatory state. As we all know, the cause of fatigue is to promote inflammatory cytokines and reduce ATP libraries (energy loss). At the same time, chronic fatigue syndrome was thought to be associated with persistent activation of nuclear factor-kappa B (NF-κB), which result in augmented nitrosative–oxidative stress, lowered ATP pools, and chronic inflammation [20–22]. On one hand, NF-κB activation induces the production of high levels of reactive oxygen species (ROS), such as superoxide, on the other hand, it induces the production of nitric oxide, leading to the production of peroxynitrite, therefore, leading to changes in cell structure and function [23]. In particular, metabolic consequences will follow, such as derangement of citric acid cycle and oxidative phosphorylation, which result in lowered ATP [23–25] and NADH/NAD redox pool [23]. In addition, ROS damage ion channels, especially calcium pumps. In this way, the intracellular free calcium level increases [26–28], like ROS, which can induce a new round of NF-κB activation [26]. Bossola et al. suggested that inflammatory factors could cause fatigue in patients receiving chronic HD [29]. Cytokines and activated immune-inflammatory pathways might cause fatigue via direct effects on the central nervous system (hypothalamus, pituitary gland) and adrenal glands, or indirectly by inducing sleep disorders, depression, or anxiety [30]. Furthermore, MHD patients suffered from long term anemia, hypertension and drug therapy, which further reduce the quality of sleep and increase the fatigue of patients [31, 32]. In addition, drug therapy including calcium-containing phosphorus binders and active vitamin D, which can lead to increased serum calcium levels, which further lead to chronic kidney disease-mineral bone disorder [33], resulting in patient discomfort and restricted activities, thus affecting the patient’s quality of life and potentially leading to fatigue.

In the current subgroup analysis, we also observed a significant positive association between serum calcium and PDF in patients aged 65 and older. To the best of our knowledge, age has previously been identified as a risk factor for PDF in MHD patients [34]. Compared with younger, older people have significantly lower metabolic capacity and less physical activity, potentially leading to calcium and phosphorus metabolism disorders and resulting in fatigue after dialysis.

This study had several strengths. First, this was the first study to provide evidence that between corrected serum calcium levels after dialysis and PDF risk. Second, the study included a relatively large sample size (610 MHD patients) compared with other studies. Third, we explored corrected serum calcium levels after dialysis and the risk of PDF, which could more precisely clarify the relationship between these factors. However, the study also had some limitations. First, it was a retrospective study conducted at a single dialysis unit, which might have led to selection bias. However, our analyses were adjusted for a large number of potential confounders to overcome the differences between the two groups. Second, this was a case–control study, with reduced ability to identify a causal association compared with cohort studies; however, it could provide clues for future follow-up studies. Third, we did not use traditional questionnaires to assess patients’ fatigue status after dialysis, but a previous study using the same approach showed that patients’ subjective feelings were an important factor affecting their clinical treatment [17]. Fourth, due to the limited availability of data, although we tried to control for some potential confounding factors, we were unable to control for other conventional risk factors, such as family genetic history, smoking, drinking, occupational exposure, 25-OH-VD status, hydration status, body fluid composition and diet. However, given that adjusting for a large number of factors had little effect on the magnitude of the associations, we considered that it was unlikely that any unmeasured or imperfectly measured factors would materially change the results. Finally, corresponding parameters in the model were not optimized before the study commencement and it will be the subject of future work.

In conclusion, the present case–control study indicated that there was a positive association between corrected serum calcium levels after dialysis and the risk of PDF. These results suggest that prevention strategies aimed at reducing corrected serum calcium levels should be implemented to decrease the risk of PDF in the future. Given the limitations of the current study design, future large prospective studies are needed to confirm our findings and to determine the mechanism responsible for the observed relationship.

## Data Availability

The datasets used and/or analyzed during the current study are available from the corresponding author upon reasonable request.

## Abbreviations

CIs: Confidence intervals
CRP: C-reactive protein
ESRD: End-stage renal disease
HD: Hemodialysis
MHD: Maintenance hemodialysis
NF-κB: Nuclear factor-kappa B
ORs: Odds ratios
PDF: Post-dialysis fatigue
ROS: Reactive oxygen species
spKt/V: Single-pool Kt/V_urea_
SD: Standard deviation
